# Transscleral cyclophotocoagulation and its histological effects on the conjunctiva

**DOI:** 10.1038/s41598-019-55102-0

**Published:** 2019-12-10

**Authors:** Nicholas Y. Q. Tan, Marcus Ang, Anita S. Y. Chan, Veluchamy A. Barathi, Clement C. Tham, Keith Barton, Chelvin C. A. Sng

**Affiliations:** 1 Singapore Eye Research Institute, Singapore, Singapore; 2 Singapore National Eye Centre, Singapore, Singapore; 3 Ophthalmology and Visual Sciences Academic Clinical Program, Duke-NUS Medical School, Singapore, Singapore; 4 Department of Ophthalmology, Yong Loo Lin School of Medicine, National University of Singapore, Singapore, Singapore; 5 Department of Ophthalmology and Visual Sciences, The Chinese University of Hong Kong, Shatin, New Territories Hong Kong SAR; 6 Hong Kong Eye Hospital, Kowloon, Hong Kong SAR; 7 Moorfields Eye Hospital, NHS Foundation Trust, London, UK; 8 Institute of Ophthalmology, University College, London, UK; 9 Ophthalmology Department, National University Hospital, Singapore, Singapore

**Keywords:** Adverse effects, Preclinical research

## Abstract

Micropulse transscleral cyclophotocoagulation (MP-TCP) is increasingly being used as an initial procedure prior to conjunctival filtration surgeries. However, it is uncertain whether MP-TCP may cause inflammation and scarring of the bulbar conjunctiva. Thus, we aimed to study the histological effects of MP-TCP (compared to controls and continuous wave [CW]-TCP) on the conjunctiva. Our study included 10 Dutch Belted Rabbits that underwent TCP in their right eyes (*n* = 5, MP-TCP; *n* = 5, CW-TCP), while their left eyes served as controls. The rabbits were euthanised at 4 weeks, and their dissected globes underwent histopathological and immunohistochemical examination. We observed greater conjunctival inflammation in MP-TCP or CW-TCP-treated eyes compared to controls, but not between each other. The majority of the lymphocytic infiltrates were CD4 T-cells. Increased conjunctival fibrosis was evident in MP-TCP or CW-TCP-treated eyes, to similar extents, compared to controls. However, the increased staining for myofibroblasts was not statistically significant in TCP-treated eyes. We concluded that MP-TCP causes significantly greater overall conjunctival inflammation and scarring compared to controls, similar to CW-TCP. As these are risk-factors for fibrosis and failure of the conjunctival bleb, further studies are required to explore the effect, if any, of post-TCP conjunctival changes on future bleb morphology and survival.

## Introduction

The ablation of the ciliary body epithelium using thermal^1^, cryo^2^ or laser^3–6^ therapy has been employed as a treatment for glaucoma since the 1930s. The aim of cyclophotocoagulation is to reduce aqueous humour production and lower the intraocular pressure (IOP)^3^. In transscleral cyclophotocoagulation (TCP), a laser beam is transmitted through the overlying sclera, and is absorbed by melanin in the ciliary processes, resulting in photocoagulation of ciliary body tissues^3–6^. Contact TCP using the continuous wave semiconductor diode laser (810 nm) laser^5,6^ (CW-TPC) is the most common mode of laser delivery in modern clinical practice due to its efficacy, cost and portability^7^. However, CW-TCP may sometimes have an unpredictable IOP-lowering effect (e.g., resulting in hypotony), and is also associated with risk of sight-threatening complications such as phthisis bulbi, scleral melt and sympathetic ophthalmia^8–13^. Thus, CW-TCP has traditionally been reserved as a treatment of last resort in refractory glaucoma, or as a non-incisional means of lowering IOP in painful blind eyes^7,14^.

In recent years, a newer technique of TCP has been developed that uses a micropulsed delivery of laser energy (MP-TCP)^15–17^. The micropulse mode administers a series of short pulses of diode laser separated by pauses. Theoretically, cyclic laser application allows energy to build up in the targeted pigment tissues, eventually reaching a phototherapeutic state; concurrently, the pauses allow adjacent non-pigmented tissues to cool down and remain below the photocoagulative threshold^18–20^. This is thought to minimise the collateral tissue damage that may be seen after CW-TCP^21,22^. MP-TCP has accordingly been shown to have an excellent safety profile, with low risk of serious complications^16,17,23–27^. For instance, in the largest study to date, in a total of 197 eyes of 161 patients treated with MP-TCP, and followed up for a median of 12 months, there were no intra-operative complications, and the only post-operative complications were 4 cases of cystoid macular oedema, which resolved without any loss of visual acuity^24^. Numerous other clinical studies in both adult and paediatric patients have also reported an absence of serious complications associated with MP-TCP^16,17,25–27^. Therefore, in clinical practice, some surgeons have utilised MP-TCP in the earlier stages of glaucoma, sometimes even before attempting glaucoma filtration surgery^24^. However, it is plausible that the application of laser energy over the ciliary body may also alter the health of the overlying bulbar conjunctiva and sclera. This may in turn affect the outcome of future subconjunctival filtration surgery (e.g. trabeculectomy, nonpenetrating deep sclerectomy, Ex-PRESS shunts, aqueous shunts, or subconjunctival microinvasive glaucoma surgery devices) if subsequently required. However, there is scant literature on the outcomes trabeculectomies after TCP. Prior TCP is an exclusion criterion for almost all major clinical trials involving trabeculectomies, as it is assumed to affect future surgical outcome. Furthermore, to the best of our knowledge, no prior studies have described the effect of MP-TCP on the perilimbal conjunctiva in either human or animal eyes. To address this gap, the following study was designed to compare the conjunctival histology after MP-TCP compared with CW-TCP and untreated control eyes in an animal model.

## Materials and Methods

Ten female Dutch Belted Rabbits (1.5–2.5 kg, 2–4 months old; Covance Research Products Inc., Denver, PA, USA) were acclimatized for 7 days before the start of the experiments. The study was carried out in accordance with the Association for Research in Vision and Ophthalmology statement for the use of animals in ophthalmic and vision research, and was approved by the SingHealth Institutional Animal Care and Use Committee.

### Transscleral photocoagulation

The right eye of all rabbits received TCP whilst the left eye served as a control. The animals were randomly allocated to one of two treatment regimens: MP-TCP or CW-TCP (*n* = 5 in each group). TCP was performed under sedation (intramuscular ketamine 50 mg/kg and xylazil 10 mg/kg), and with topical (tetracaine hydrochloride 1.0%) and peribulbar (lignocaine hydrochloride 2%) anesthesia. A lid speculum was used for optimal exposure.

MP-TCP was applied using the Cyclo G6 Glaucoma Laser System with the MicroPulse P3 Glaucoma Device (IRIDEX Laser Systems, Mountain View, CA, USA). Settings were adjusted to 2000 mW of 810 nm infrared diode laser applied for 100 s treatment time with a duty cycle of 31.3%; this consisted of 62,500 micropulses during which the laser was “ON” for 0.5 ms and “OFF” for 1.1 ms, delivering 62.6 J of energy in total. The front edge of the cycloprobe was adjusted at the limbus, perpendicular to the sclera, such that the centre of the fibre tip was directed towards the pars plana (it is hypothesized that MP-TCP’s IOP-lowering effects work via local tissue remodelling on the pars plana, and the increase of uveoscleral aqueous outflow^28^). We avoided the 3 and 9 o’clock meridians where the ciliary neurovascular bundles were located.

CW-TCP was applied using the same Cyclo G6 Glaucoma Laser System with a G probe (IRIDEX Laser Systems, Mountain View, CA, USA) with the tip removed, and the laser fibre optic filament positioned just posterior to the limbus, targeting the pars plicata^21,29^. Continuous diode laser was titrated around 2000 mW (when an audible ‘pop’ was heard, signifying a uveal micro-explosion, the power was reduced by 250 mW for the rest of the treatment^30^), with 2 s exposure time per burn, and 16 burns per eye, delivering approximately 64 J of energy in total.

Following TCP, topical prednisolone acetate 1% eye drops were administered four times daily for 1 week to the treatment eye, and then tapered to twice daily for 2 weeks.

IOP was measured with a handheld tonometer (Tonopen; Mentor, Norwell, MA) after topical anaesthesia (tetracaine hydrochloride 1.0%) at baseline and at post-treatment weeks 1, 2, 3 and 4. At each measurement, 4 readings were taken per eye, and the mean result was used. An examiner masked to the treatment received carried out the IOP measurements.

### Histological evaluation

The rabbits were euthanised with intraperitoneal pentobarbitone (100 mg/kg) at the end of the study period of 4 weeks. Both treatment and control eyes were enucleated and immediately immersed in a mixture of 4% paraformaldehyde and 2.5% neutral buffered formalin for 24 hours. The globes were then dissected to anterior and posterior segments, and dehydrated in increasing concentration of ethanol, cleared in xylene, and embedded in paraffin. Six-micron sections from each eye were cut with a rotary microtome and collected on microscope glass slides. The sections were dried in an oven of 37 °C for at least 24 hours. To prepare the sections for histopathological and immunohistochemical examination, the sections were heated on a 60 °C heat plate, deparaffinized in xylene and rehydrated in decreasing concentration of ethanol. Histological evaluations were conducted by A.S.Y.C. who was blinded to what treatment each eye had received.

A standard procedure for Hematoxylin and Eosin (H&E) was performed to assess total cellularity and inflammatory cells (macrophages and leucocytes). Picro-Sirius Red (Abcam ab150681, Cambridge, MA, USA) and Masson’s trichrome staining (26367-Series, Electron Microscopy Sciences) were also performed to stain for collagen fibres and evaluate conjunctival tissue fibrosis. A light microscope (Axioplan 2; Carl Zeiss Meditec GmbH, Oberkochen, Germany) was used to examine the slides and images were digitally captured.

For estimating the amount of conjunctival inflammation and conjunctival scarring, a scoring system (modified from that described by Shah *et al*.^31^) was defined as Grade 0 = no inflammation/fibrosis; 1 = mild inflammation/fibrosis; 2: moderate inflammation/fibrosis; and 3 = severe inflammation/fibrosis. This was based on visual reference standards for grades from 0 to 3: 0 = same as control eye; 1 = 1%–33% of control; 2 = 33%–66% of control; 3 = more than 66% of control.

In parallel, immunofluorescence (IF) staining was performed to ascertain the nature of the infiltrating cells. Details of the antibodies used are included in Table 1. Smooth muscle actin was chosen to identify myofibroblast to determine cells that might account for collagen deposition. Pan-leukocyte marker, or leukocyte common antigen (LCA), that identifies all lymphocytes including monocytes and eosinophils, and CD4 T cell marker, were also used to identify the nature of inflammatory infiltrates. In brief, our IF protocol includes heat-induced antigen retrieval performed by incubating sections in sodium citrate buffer (10 mM Sodium citrate, 0.05% Tween 20, pH 6.0) for 20 minutes at 95–100 °C. The sections were then cooled down in sodium citrate buffer for 20 minutes in room temperature and washed three times for 5 minutes each with 1x phosphate-buffered saline (PBS). Non-specific sites were blocked with blocking solution of 5% bovine serum albumin in 0.1% Triton X-100 and 1x PBS for 1 hour at room temperature in a humidified chamber. The slides were then rinsed briefly with 1x PBS. A specific smooth muscle actin antibody shown in Table 1 was applied and incubated overnight at 4 °C in a humidified chamber prepared in blocking solution. After washing twice with 1x PBS and once with 1x PBS with 0.1% tween for 10 minutes each, Alexa Fluro® 488 – conjugated fluorescein-labeled secondary antibody shown in Table 1 (Invitrogen- Molecular Probes, Eugene, OR) was applied at a concentration of 1:1000 in blocking solution and incubated for 90 minutes at room temperature. In conjunction, a cocktail set of CD4 and LCA antibodies also shown in Table 1 was applied and incubated similarly with corresponding secondary antibodies (conjugated with Alexa 488 and Alexa 594; Molecular Probes, Eugene, OR, USA). The slides were then washed twice with 1x PBS, and once with 1x PBS with 0.1% Tween 20 detergent for 5 minutes each, the slides were mounted on the slides with Prolong Diamond Anti-fade DAPI5 Mounting Media (Invitrogen- Molecular Probes, Eugene, OR) to visualize cell nucleic. For negative controls, primary antibody was omitted. A fluorescence microscope (Axioplan 2; Carl Zeiss Meditec GmbH, Oberkochen, Germany) was used to examine the slides and images were captured. Experiments were repeated in duplicates for the antibody.

**Table 1:** Immunohistochemistry stains.

Antibody	Catalog No.	Company	Concentration
CD4	553043	BD Pharmigen (Franklin Lakes, NJ)	1:50
Leukocyte common antigen	sc-70690	Santa Cruz (Santa Cruz Biotechnology, Santa Cruz, CA)	1:50
Smooth actin muscle	Ab7817	Abcam (Cambridge, MA, USA)	1:400
Alexa Fluor 488 goat anti−mouse IgG (H + L)	A11006	Invitrogen. Life Technologies (Invitrogen, Eugene, OR)	1:1000
Alexa Fluor 488 goat anti−rat IgG (H + L)	A11032	Invitrogen. Life Technologies (Invitrogen, Eugene, OR)	1:1000

For evaluation of CD4, LCA and Smooth Actin Muscle (SMA) IF, the number of positively-stained cells was counted within one high power field (HPF) magnification (40x magnification, 0.55 mm field diameter, Olympus microscope), and a semiquantitative grading scale was used: 0 = no positive cells staining; 1 = mild infiltrate of cells (<10 positive cells per 1 high power field [HPF]); 2 = moderate infiltrate of cells (10–20 cells per HPF); and 3 = severe infiltrate of cells (>20 cells per HPF).

### Statistical analysis

Statistical analysis was performed using IBM SPSS Statistics for Macintosh, Version 25.0 (IBM Corp., Armonk, NY, USA). Normality of data was assessed using the Shapiro-Wilk test. For the normally distributed IOP readings, the one-way analysis of variance (ANOVA) was used to determine whether there were any statistically significant differences between the means of the 3 treatment groups (controls, CW-TCP and MP-TCP). To assess whether any of the 2 groups’ mean IOPs differed from each other, an independent t-test was performed. Linear regression was used to estimate the IOP-lowering effect of MP-TCP and CW-TCP compared to controls; in doing so, a generalised estimating equation was employed to account for the correlation between eyes within rabbits, and also the correlation of repeated IOP-measurements within eyes. For the graded inflammatory and fibrotic changes of the conjunctiva assessed on histology, these parameters were non-normally distributed, and hence the nonparametric Kruskal-Wallis test and the Mann-Whitney U test were performed to compare between the 3 treatment groups, or any of the 2 treatment groups, respectively. *P* value for significance was set at <0.05.

## Results

The mean IOPs at each assessment time point are summarised in Table 2. In all eyes treated with TCP, there was a significant reduction of IOP compared to control eyes sustained throughout all 4 weeks (all *P* ≤ 0.01). However, between eyes treated with CW-TCP and MP-TCP, there were no significant differences in mean IOP between the 2 groups. Compared to control eyes, eyes treated with MP-TCP and CW-TCP were associated with significant reduction in IOP (all *P* ≤ 0.01) of −7.1 (95% confidence interval [CI]: −5.2 to −8.8) and −6.3 (95% CI: −5.0 to −8.8) mmHg, respectively. There was no significant difference in the IOP-lowering effect between MP-TCP and CW-TCP (*P* = 0.34). No eyes were hypotonous (IOP ≤ 5 mmHg) at any assessment time point.

**Table 2:** The intraocular pressure in transscleral cyclophotocoagulation-treated eyes and control eyes at baseline and post-treatment.

Timepoint	IOP			P^a^	P^b^	P^c^	P^d^
	Controls	CW-TCP	MP-TCP				
Baseline	19.5 (2.5)	17.2 (0.7)	18.1 (1.9)	0.16	0.08	0.31	0.39
Week 1	20.3 (5.1)	12.4 (4.6)	7.6 (2.1)	**<0.001**	**0.01**	**<0.001**	0.07
Week 2	12.8 (0.7)	7.4 (1.1)	7.1 (1.3)	**<0.001**	**<0.001**	**<0.001**	0.72
Week 3	13.8 (1.3)	8.3 (0.9)	7.9 (1.4)	**<0.001**	**<0.001**	**<0.001**	0.63
Week 4	16.2 (1.6)	10.0 (1.5)	12.2 (4.0)	**<0.001**	**<0.001**	**0.01**	0.29

CW-TCP: continuous wave transscleral cyclophotocoagulation; IOP: intraocular pressure; MP-TCP: micropulse transscleral cyclophotocoagulation.

Data are presented as mean (standard deviation).

^a^P value for the difference between the 3 groups (controls, CW-TCP and MP-TCP).

^b^P value for the difference between the controls and CW-TCP.

^c^P value for the difference between the controls and MP-TCP.

^d^P value for the difference between the MP-TCP and CW-TCP.

**Figure 1:**
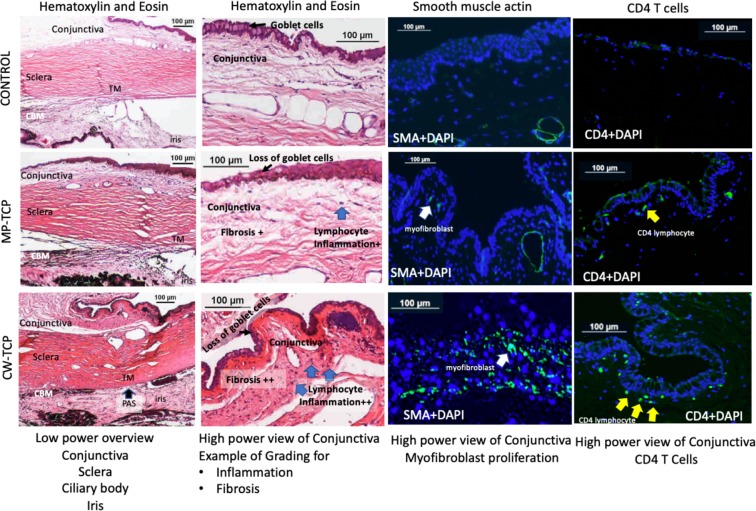
Histological images of the conjunctiva under light microscopy after staining with Hematoxylin and Eosin (first two columns), and for immunohistochemistry for smooth muscle actin (third column) and CD4 T cells (fourth column). A representative eye from each treatment group (controls: top row; MP-TCP: middle row; and CW-TCP: bottom row) are shown. The MP-TCP-treated eye had milder inflammation and less fibrosis of the bulbar conjunctiva compared to the CW-TCP-treated eye, which also demonstrated peripheral anterior synechiae and haemorrhage in this specimen. On immunofluorescence, staining for SMA and CD4 T cells was stronger in the eye treated with CW-TCP compared to MP-TCP. However, the difference in the average inflammation and fibrosis between CW-TCP and MP-TCP in all eyes was not statistically different (Tables 3 and 4). CBM, Ciliary body muscle; CW-TCP: continuous wave transscleral cyclophotocoagulation; MP-TCP: micropulse transscleral cyclophotocoagulation; PAS, Peripheral anterior synechiae; TM, Trabecular Meshwork.

**Figure 2:**
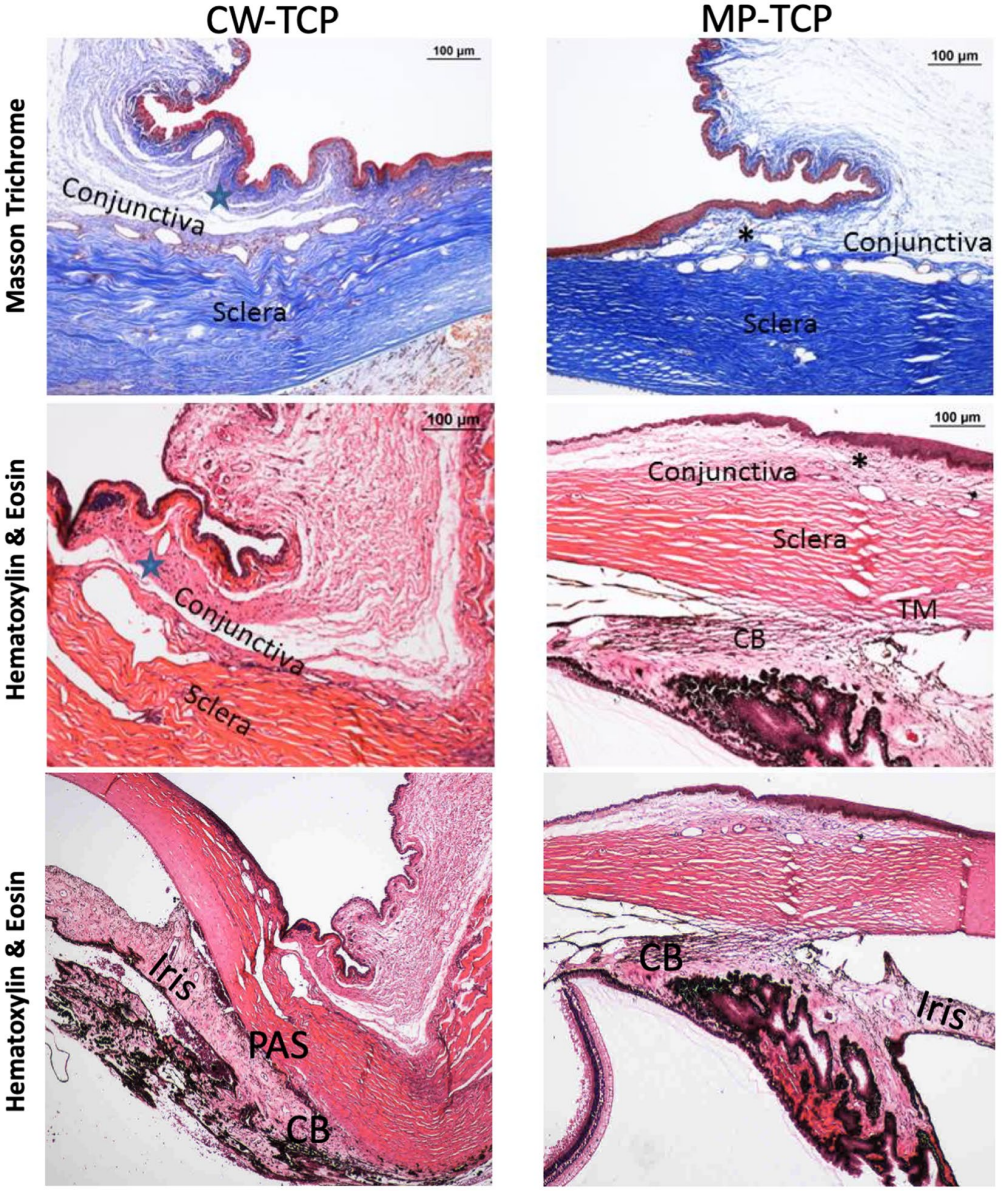
Histological images of the conjunctiva (first two rows) and ciliary body (last row) under light microscopy after staining with Masson trichrome (first row) and Hematoxylin and Eosin (last two rows). A representative eye that underwent CW-TCP (left column) MP-TCP (right column) are shown. CBM damage and PAS were found in the CW-TCP eye in contrast to the open angle and relatively preserved CBM anatomy seen in MP-TCP. Masson trichrome blue staining highlights greater conjunctival fibrosis seen in the eye treated with CW-TCP (blue star) compared to MP-TCP (asterix). However, the difference in the average inflammation and fibrosis between CW-TCP and MP-TCP in all eyes was not statistically different (Tables 3 and 4). CBM, Ciliary body muscle; CW-TCP: continuous wave transscleral cyclophotocoagulation; MP-TCP: micropulse transscleral cyclophotocoagulation; PAS, Peripheral anterior synechiae; TM, Trabecular Meshwork.

The effects of TCP on the conjunctiva and ciliary body were evaluated histologically at the end of 4 weeks. Representative histological images focusing on the conjunctiva and ciliary body are shown in Figs. 1 and 2. A number of TCP-treated eyes had loss of goblet cells in the conjunctival epithelium, and an increase in conjunctival stromal fibrosis observed on H&E staining (Fig. 1) and Masson’s trichrome staining (Fig. 2) that stained for collagen fibres. In some eyes, we also found that MP-TCP resulted in milder inflammation and less fibrosis of the bulbar conjunctiva and ciliary body compared to CW-TCP.

**Figure 3:**
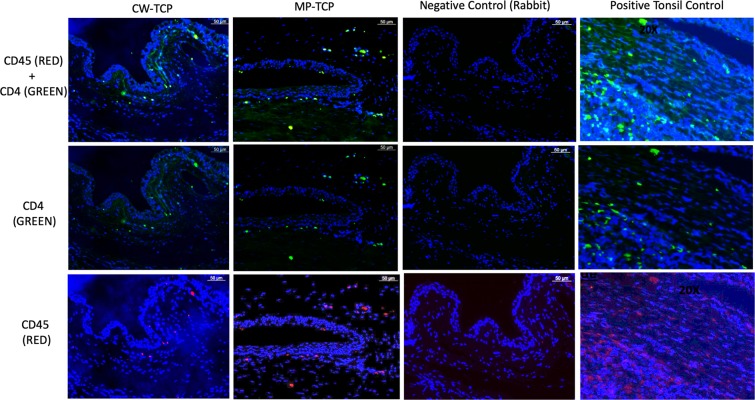
CD45 pan-leucocyte marker and CD4-T cells on immunofluorescence show co-localization of both markers indicating that the predominant inflammation cell type is CD4-positive T cells.

However, when these qualitative histological changes in the conjunctiva and ciliary body were quantified (Tables 3 and 4, respectively), the effects of MP-TCP and CW-TCP on the rabbit eye were similar. Overall, compared to controls, eyes treated with both types of TCP had more inflamed conjunctivas and ciliary bodies when assessed grossly for inflammatory cell infiltration on H&E staining, and on immunohistochemistry for a semiquantitative count of CD4 and LCA reactivity for T-cells and pan-leucocyte, respectively. However, there were no significant differences in inflammation post-MP-TCP or post-CW-TCP. Notably, the semiquantitative counts of positively-stained cells for CD4 and LCA were strongly correlated with one another (Pearson correlation coefficient = 0.94; *P* < 0.001), suggesting that majority of the lymphocytic infiltrates identified were CD4 T-cells. Accordingly, double staining with CD4 and LCA in different colours show that the predominant cell type was CD4-positive  as it entirely colocalises with CD45 (Fig. 3).

**Table 3:** The conjunctival changes in transscleral cyclophotocoagulation-treated eyes and control eyes at the end of 4 weeks.

Conjunctival histology	Controls	CW-TCP	MP-TCP	P^c^	P^d^	P^e^	P^f^
**Inflammation**							
Gross inflammation on Hematoxylin & Eosin stain^a^	0 (0)	1.4 (0.6)	1.4 (0.9)	**0.001**	**0.001**	**0.013**	0.84
T cells using CD4 immunohistochemistry^b^	0.1 (0.2)	0.9 (0.3)	0.9 (0.5)	**0.003**	**0.004**	**0.008**	0.89
Pan-leucocyte marker using LCA immunohistochemistry^b^	0.1 (0.2)	0.9 (0.6)	1.0 (0.6)	**0.010**	**0.048**	**0.008**	0.89
**Fibrosis**							
Gross fibrosis on Masson’s trichrome stain^a^	0 (0)	2.2 (0.8)	1.8 (0.4)	**<0.001**	**0.001**	**0.001**	0.42
Myofibroblasts on Smooth Actin Muscle immunohistochemistry^b^	0.1 (0.2)	0.3 (0.3)	0.4 (0.3)	0.11	0.37	0.11	0.69

CW-TCP: continuous wave transscleral cyclophotocoagulation; MP-TCP: micropulse transscleral cyclophotocoagulation.

Data are presented as mean (standard deviation).

^a^Gross inflammation/fibrosis was graded from a scale of 0–3. Grade 0 = no inflammation/fibrosis; 1 = mild inflammation/fibrosis; 2: moderate inflammation/fibrosis; and 3 = severe inflammation/fibrosis.

^b^Immunohistochemistry inflammation/fibrosis was graded from a scale of 0–3. Grade = 0 = no positive cells staining; 1 = mild infiltrate of cells; 2 = moderate infiltrate of cells; and 3 = severe infiltrate of cells.

^c^P value for the difference between the 3 groups (controls, CW-TCP and MP-TCP).

^d^P value for the difference between the controls and CW-TCP.

^e^P value for the difference between the controls and MP-TCP.

^f^P value for the difference between the MP-TCP and CW-TCP.

**Table 4:** The ciliary body changes in transscleral cyclophotocoagulation-treated eyes and control eyes at the end of 4 weeks.

Ciliary body histology	Controls	CW-TCP	MP-TCP	P^b^	P^c^	P^d^	P^e^
Gross inflammation on Hematoxylin & Eosin stain^a^	0 (0)	1.4 (0.6)	1.4 (0.9)	0.001	0.001	0.013	0.84
Gross fibrosis of the ciliary body on Masson’s trichrome stain^a^	0 (0)	2.4 (0.5)	2.8 (0.4)	<0.001	0.001	0.001	0.31

CW-TCP: continuous wave transscleral cyclophotocoagulation; MP-TCP: micropulse transscleral cyclophotocoagulation.

Data are presented as mean (standard deviation).

^a^Gross inflammation/fibrosis was graded from a scale of 0–3. Grade 0 = no inflammation/fibrosis; 1 = mild inflammation/fibrosis; 2: moderate inflammation/fibrosis; and 3 = severe inflammation/fibrosis.

^b^P value for the difference between the 3 groups (controls, CW-TCP and MP-TCP).

^c^P value for the difference between the controls and CW-TCP.

^d^P value for the difference between the controls and MP-TCP.

^e^P value for the difference between the MP-TCP and CW-TCP.

TCP-treated eyes also had greater conjunctival and ciliary body fibrosis seen on Masson’s trichrome stain that stained for collagen fibres compared to controls. This was similar for both MP-TCP and CW-TCP. However, on staining for conjunctival myofibroblasts using Smooth Actin Muscle immunohistochemistry, although eyes treated with CW-TCP and MP-TCP had higher grades of staining compared to controls (mean [standard deviation]: 0.3 [0.3], 0.4 [0.3] and 0.1 [0.2], respectively), these differences between groups were not significant (all *P* > 0.11).

## Discussion

Histological analysis of the conjunctiva after MP-TCP has not been previously reported. Although clinical studies have demonstrated that MP-TCP has a superior safety profile with minimal risks of sight-threatening complications, and clinically evident conjunctival burns have not been reported in the literature (unlike CW-TCP^6,32,33^), it is unknown if MP-TCP may cause subclinical conjunctival damage. In our study, we used both published semi-quantitative measurements (CD4 inflammatory and myofibroblast cell counts using IF) and qualitative assessments of histology (using masson trichrome and H&E stains) to assess the degree of inflammation and fibrosis post-TCP. We have provided novel data showing inflammation and fibrosis of the conjunctiva 4 weeks after MP-TCP in rabbit eyes, at levels comparable to that of CW-TCP. Although both MP-TCP and CW-TCP had comparable IOP-lowering effects—which is consistent with prior clinical studies^17^—the conjunctival changes post-MP-TCP may potentially limit its utility as a primary treatment option in glaucoma (as it may have subsequent effects on future bleb morphology and survival).

Despite the small number of eyes included in this study, there was a clear difference in conjunctival inflammation between controls and TCP-treated eyes, but not between the MP-TCP or CW-TCP groups, on both LCA and CD4 IF staining. Notably, the grading for IF staining of LCA and CD4 were remarkably similar across all 3 groups (Table 3) and were strongly correlated with each other. LCA and CD4 co-localisation also demonstrated that majority of the inflammatory infiltrate were CD4 T helper cells. CD4 T cells are involved in the wound healing response, and have also been implicated in pathogenesis of conjunctival scarring in conjunctival cicatrizing disorders such as trachoma and ocular pemphigoid^34,35^. CD4 T helper lymphocytes are also involved in pulmonary fibrosis where the various subsets of CD4 lymphocytes can secrete profibrotic cytokines that may induce fibroblast differentiation^36^ and contribute to pathological fibrosis.

Accordingly, we have used SMA IF to detect and quantify stromal myofibroblasts, which may be recruited to lay down new collagen. On qualitative evaluation of individual eyes, we occasionally found increased conjunctival inflammation and fibrosis of the bulbar conjunctiva that appeared associated to the amount of TCP induced ciliary body damage. However, on quantitative analysis, although SMA positive myofibroblasts were present at higher levels in the stroma of both TCP groups compared to controls, this difference was not statistically significant (Table 3). Thus, although the occurrence of fibrosis may plausibly be secondary to the inflammatory infiltrate in the conjunctiva that is triggered in response to the underlying TCP-induced ciliary body damage, it may be not the only mechanism for fibrosis of the conjunctiva post-TCP. A direct burn effect, for instance, may also be contributory.

In our study, conjunctival and ciliary body inflammation and scarring were similar in MP-TCP and CW-TCP eyes. This may be a function of the amount of laser energy applied to the rabbit eyes in our treatment protocol, which was designed to be similar in both TCP-groups (so as to allow for a comparison between both modalities of treatment). For instance, in live human eyes, MP-TCP has also been associated with persistent inflammation when longer duration of laser (~300 s or more) was applied^37^.

The histological conjunctival changes that we have found post-MP-TCP (and CW-TCP) may have clinical implications. Although MP-TCP was first developed as a newer iteration of TCP for its established use in refractory glaucoma, its improved safety profile has resulted in MP-TCP being performed on eyes with less advanced glaucoma as an initial procedure prior to conjunctival filtration surgeries^24^. However, on histology, both CW-TCP and MP-TCP resulted in conjunctival inflammation and scarring. This is concerning, as prior studies demonstrated that eyes at higher risk of failure after filtration surgery had increased conjunctival inflammatory cells and fibroblasts^38–40^. As a primary procedure, TCP is less efficacious than trabeculectomy in lowering IOP^41^. Hence, we would advise caution in offering MP-TCP (or CW-TCP) as an primary intervention for patients with mild-to-moderate glaucoma, if there is a possibility of trabeculectomy still being needed down the line. However, if MP-TCP is performed prior to trabeculectomy (for instance, to control IOP pre-phacotrabeculectomy in medically unresponsive acute primary angle closure eyes)^42^, it may be prudent to spare the superior conjunctiva in case future trabeculectomy is subsequently required^42^. The role of an intermediate procedure in the glaucoma treatment paradigm—considering the detrimental effects TCP may have on the conjunctiva—may therefore potentially be better filled by other treatment options including selective laser trabeculopasty^43^ and variants of microinvasive glaucoma surgery that spare the conjunctiva^44^.

There are limitations to this study. First, the rabbit sclera is approximately half as thick as the human sclera—i.e., there is a shorter distance between the ciliary body (the target of laser energy) and the conjunctiva (where collateral direct damage may arise) in rabbits compared to humans^45^. Our results may therefore be biased towards greater conjunctival changes due to the difference in ocular anatomy in rabbits. Second, our sample size was small; this may have limited our study’s ability to statistically compare the small differences in the amount of inflammation/scarring between MP-TCP- and CW-TCP-treated eyes. Third, because our experiments were performed in normal and previously untreated eyes, the results attained may not be the same in eyes with glaucoma treated with topical IOP-lowering medications. Topical antiglaucoma agents have been shown to cause conjunctival cellular “activation” that accelerates scar formation in blebs following glaucoma filtration surgery^39,40,46^. If the effect of MP-TCP on conjunctival scarring is additive or multiplicative with topical glaucoma medications^47^, this may be an even stronger reason to avoid TCP before attempting trabeculectomy. Fourth, although our gradings were done by a qualified pathologist (A.S.Y.C.) who was masked to the treatment groups, we did not have a second grader. Fifth, although we have shown statistically significant histological changes in the conjunctiva of MP-TCP-treated eyes compared to controls, the clinical significance of such changes will need to be verified in further studies. This involves studying the potential effect of MP-TCP on future conjunctival blebs, as well as comparing it with the effect from the usage of topical IOP-lowering medications.

In conclusion, in the healthy eyes of pigmented Dutch Belted rabbits, both MP-TCP and CW-TCP cause significant perilimbal conjunctival inflammation and scarring on histological analysis. These cellular changes may increase the risk of bleb fibrosis and failure after filtration surgery, and hence undermine the utility of MP-TCP as an initial treatment option, especially in patients with mild-to-moderate glaucoma. Further studies are required to explore the clinical consequence of post-MP-TCP conjunctival changes on bleb morphology and survival.

## Data Availability

The data that support the findings of this study are available from the corresponding author, upon reasonable request.
